# Emergence of a *Streptococcus dysgalactiae* subspecies *equisimilis stG62647*-lineage associated with severe clinical manifestations

**DOI:** 10.1038/s41598-017-08162-z

**Published:** 2017-08-08

**Authors:** Oddvar Oppegaard, Haima Mylvaganam, Steinar Skrede, Paul Christoffer Lindemann, Bård Reiakvam Kittang

**Affiliations:** 1 Department of Medicine, Haukeland University Hospital, Bergen, Norway; 2 Department of Clinical Science, University of Bergen, Bergen, Norway; 3 Department of Microbiology, Haukeland University Hospital, Bergen, Norway; 4 Department of Medicine, Haraldsplass Deaconess Hospital, Bergen, Norway

## Abstract

Increasing incidence rates of invasive *Streptococcus dysgalactiae* subspecies *equisimilis* (SDSE) infections have been reported worldwide, but the evolutionary mechanisms underlying this development remain elusive. Through prospective surveillance of invasive SDSE infections in western Norway, we observed the emergence of a novel and virulent SDSE genotype, *stG62647*. This *emm*-type, rarely encountered as a cause of invasive disease during 1999–2012, emerged in 2013 as the predominant SDSE-genotype. The *stG62647*-infections were associated with an aggressive clinical course, including the occurrence of streptococcal toxic shock syndrome, necrotizing soft-tissue infections and endocarditis. All the invasive *stG62647*-isolates were subjected to whole genome sequencing, attempting to explore the genetic events underpinning its epidemicity. Although 10% of the genomes was unique for *stG62647*-genotype, notably 18 out of 19 isolates contained a disrupted *streptococcal invasive locus* (*sil*) due to the insertion of a transposase, IS1548, into the *silB*-gene. We postulate that the virulence of *stG6267*-isolates could be partly attributable to the abrogation of the attenuating control normally exerted by this regulon, although experimental verification was not performed. To the best of our knowledge, this is the first study employing large scale whole genome sequencing to illuminate the genetic landscape of epidemic lineages in SDSE.

## Introduction

*Streptococcus dysgalactiae* subspecies *equisimilis* (SDSE) is an emerging human pathogen with a disease spectrum similar to the closely related *Streptococcus pyogenes* (*S. pyogenes*)^1, 2^. Although SDSE has been regarded as less virulent than *S. pyogenes*, severe clinical manifestations have been documented, including necrotizing soft tissue infection and streptococcal toxic shock syndrome^3, 4^. SDSE and *S. pyogenes* also share extensive genetic homology, including genes encoding conserved virulence factors (*emm*, *streptolysin O*, *streptolysin S*, *streptokinase* and *c5a peptidase*) and virulence regulators (*control of virulence*-regulon [*covRS*] and *streptococcal invasive locus* [*sil*])^5, 6^.

During the past decade, an upsurge in the incidence of invasive SDSE disease has been documented worldwide, in some geographic regions even surpassing the incidence of invasive *S. pyogenes* infections^7–9^. In our region of western Norway, SDSE is currently the leading cause of invasive beta-haemolytic disease^2^. The epidemiological and molecular events promoting this development, however, have yet to be elucidated. Several studies involving whole genome sequencing have investigated the genomic events leading to epidemic spread of hypervirulent *S. pyogenes* clones, revealing mutations in virulence regulators and virulence genes, as well as the acquisition of novel bacteriophages^10, 11^. Similar studies have not been conducted for SDSE.

Mutations in regulons normally exerting negative transcriptional control of virulence genes have been demonstrated to induce the transition from a mild to a more invasive phenotype, particularly in the *covRS*-regulon and the *sil*-locus^12, 13^. *covRS* is a two-component regulatory system, comprising the genes *covR* and *covS*, controlling the expression of approximately 10% of the *S. pyogenes* transcriptome^14^. *covRS*-mutants have an upregulated expression of many virulence factors, increased resistance to phagocytosis, and increased virulence potential in murine bacteraemia models^14^. *sil* is a virulence-regulon found in approximately 80% of SDSE and 25% of *S. pyogenes*, and *S. pyogenes*-isolates with mutations in this locus have been reported to cause infections pursuing an aggressive clinical course^6, 13^. *sil* comprises genes encoding a putative two component regulator (*silA* and *silB*), a transporter system (*silD* and *silE*), and two small pheromone-like peptides (*silC* and *silCR*) with differing functions; *silC* being linked to virulence, whereas *silCR* is associated with competence, bacterial quorum sensing, and attenuation of virulence in both SDSE and *S. pyogenes*-infections^15^.

Through prospective registration and genotyping of invasive SDSE-disease from 2012, we identified an outbreak of the SDSE *emm*-type *stG62647* in our health region. Here, we present a detailed epidemiological and molecular characterization of the outbreak, and through whole genome sequencing we attempted to unravel the genetic rationale for this virulent genotype, with particular emphasis on acquired bacteriophages, the repertoire of virulence factors, and mutations in the virulence regulons *covRS* and *sil*.

## Materials and Methods

### Study setting and definitions

Health Region Bergen in Western Norway has a catchment area of 430 000 inhabitants, and comprises the tertiary care hospital Haukeland University Hospital, along with the two secondary care hospitals Haraldsplass Deaconess Hospital and Voss Hospital. Data on the epidemiology of SDSE-disease in this region in the period 1999–2013 has previously been reported^2^. From 2012 to 2015 SDSE isolates obtained from invasive disease were prospectively collected, and clinical information was abstracted from patient records. All *stG62647*-isolates during 1999–2015 were included in the study. Invasive disease was defined as isolation of SDSE from a sterile site, or from a non-sterile site in association with verified necrotizing soft tissue infections. Clinical entities and streptococcal toxic shock syndrome were defined as previously described^2^. Severe disease was defined as the presence of necrotizing soft tissue infection, endocarditis, streptococcal toxic shock syndrome or death ensuing within 30 days of admission.

### Bacterial isolates, DNA extraction and sequencing

All isolates displayed large colony size (>0.5 mm in diameter after 24 hours) and β-haemolysis on 5% sheep blood agar. Serogroup specificity had previously been determined using a rapid agglutination test (Oxoid Streptococcal Grouping Kit, Hampshire, UK), and species identity had been confirmed by matrix-assisted laser desorption ionization-time of flight mass spectrometry (MALDI-ToF MS), using Microflex™ with the MALDI Biotyper database (Bruker Daltonik, Bremen, Germany). Bacterial suspensions were pre-treated with Mutanolysin (M9901, Sigma-Aldrich, Dorset, UK), Lysozyme (L6876, Sigma-Aldrich) and Proteinase K (19133, Qiagen, Hilden, Germany), and genomic DNA was extracted using MagNA Pure 96 (Roche Life science). Genomic libraries were made using Nextera XT Kit (Illumina, Essex, UK), and 150 basepairs paired-end sequencing was performed on HiSeq. 4000 (Illumina).

### Assembly, annotation and molecular characterization

Reads quality was assessed using FastQC (bioinformatics.bbsrc.ac.uk), and filtered using Trimmomatic^16^. Trimmed reads were submitted to Center for Genomic Epidemiology for online assembly by Spades^17^, and subsequent assessment with Quast^18^. Annotation was performed using RAST^19^. *emm*-type was determined by BLAST-search against the *emm*-type database curated by Centers for Disease Control (http://www.cdc.gov). Multilocus sequence typing (MLST) profiles were identified using the Center for Genomic Epidemiology website^20^, and novel MLST alleles and profiles were submitted to pubMLST (www.pubMLST.org). To compare the strains for variations across the whole genome, a single nucleotide polymorphism (SNP) based phylogenetic analysis was performed as previously described^21^. Of the six full genomes deposited in GenBank, *S. dysgalactiae* subspecies *equisimilis* ATCC 12394 (GenBank accession number CP002215) had the closest genetic resemblance to our *stG62647*-genomes, and was selected as a reference-genome in the SNP-analysis. Geneious version 10.0 was used to screen for the presence and mutation of selected virulence genes and regulators (Table 1)^22^. The selection of genes was adapted from the *S. pyogenes*-virulence factors listed in the Virulence Factors Database (www.mgc.ac.cn/VFs), with some minor modifications. Genomes were screened for bacteriophages by Phaster, and for Clustered Regularly Interspaced Short Palindromic Repeats (CRISPRs) using CRIPSRFinder^23, 24^.

**Table 1:** Genetic repertoire of the invasive *stG62647*-isolates.

Gene	Reference	CC20	ST17	Phage	Rep. SDSE
**Adhesion**					
*dltABCD-*operon	BAH81801–4	**+**	**+**	—	**+**
*ProteinF1/gfba*	U31115	—	**+**	—	**+**
*ProteinF2/fbaB/PFBP*	AB084272/AY612221	—	—	—	**−**
*fbaA*	AB040536	—	—	—	**+**
*Sof*	X83303	—	—	—	**−**
*sfbX*	AF335322	—	—	—	**−**
*gapC*	AF375662	**+**	**+**	—	**+**
*fbp54*	BAH81758	**+**	**+**	—	**+**
*Shr*	BAH82346	**+**	**+**	—	**+**
*Lmb*	BAH81434	**+**	**+**	—	**+**
*sclA*	AY459361				
**Enzymes**					
*Streptolysin O*	AB050249	**+**	**+**	—	**+**
*Streptolysin S*	AY033399	**+**	**+**	—	**+**
*Streptokinase*	BAH80750	**+**	**+**	—	**+**
*Streptodornase (sda)*	AAL98274	—	—	**+**	—
*Streptodornase B (sdaB)*	AAM80352	—	—	**−**	—
*Streptodornase D (sdaD)*	WP_048327803	—	—	**+**	**+**
*Streptodornase (sdn)*	BAH81602	—	—	**+**	**+**
*Mitogenic factor 2*	AAL97446	—	—	**+**	—
*Mitogenic factor 3*	KKC20252	—	—	**+**	**+**
*Mitogenic factor 4*	AAM79702	—	—	**+**	—
*Phospholipase A2*	AAM79811	—	—	**+**	**+**
**Immune evasion**					
*drsG*	BAH81431	—	**+**	—	**+**
*Protein G*	M13825	**+**	**+**	—	**+**
*C5a-peptidase*	BAH81432	**+**	**+**	—	**+**
*hasA*	WP_011055113	—	—	—	—
*hasB*	WP_002992300	—	—	—	—
*hasC*	BAH82462	**+**	**+**	—	**+**
**Superantigens**					
*speA*	U40453	—	—	**+**	**+**
*speB (protease)*	L26125	—	—	—	—
*speC*	M35514	—	—	**+**	**+**
*speG*	AB105080	**+**	—	—	**+**
*speH*	AF124500	—	—	**+**	**+**
*speI*	AF438524	—	—	**+**	**+**
*speJ*	AF321000	—	—	—	—
*speK*	WP_011054728	—	—	**+**	—
*speL*	WP_011017837	—	—	**+**	—
*speM*	AIT77602	—	—	**+**	**+**
*Ssa*	U48792	—	—	**+**	**−**
*smeZ*	AB046865	—	—	—	**+**
**Virulence regulon**					
*covRS*	BAH80897/8	**+**	**+**	—	**+**
*Sil*	GQ184566	Mut	—	—	+

CC20, *stG62647*-isolates belonging to clonal complex 20; ST17, *stG62647*-isolate belonging to MLST-profile 17; Phage, bacteriophage-associated; Rep. SDSE, previously reported in SDSE; Mut, mutated. Symbols + and–denote presence or absence of genetic feature, respectively.

### Statistical analysis

Data were processed using SPSS PASW STATISTICS, version 21.0 (IBM SPSS Statistics for Windows, Version 21.0. Armonk, NY: IBM Corp). Categorical data were analysed using Fisher’s exact test, and non-parametric data were analysed with Mann Whitney U-test. A two-sided p-value ≤ 0.05 was considered statistically significant.

### Ethical standards

The study underwent institutional ethics review and approval (2010/1406 Regional Ethics Committee West, Norway). Informed consent was obtained from participants. All experiments were carried out in full accordance with the approved ethics applications specified above.

## Results

**Figure 1:**
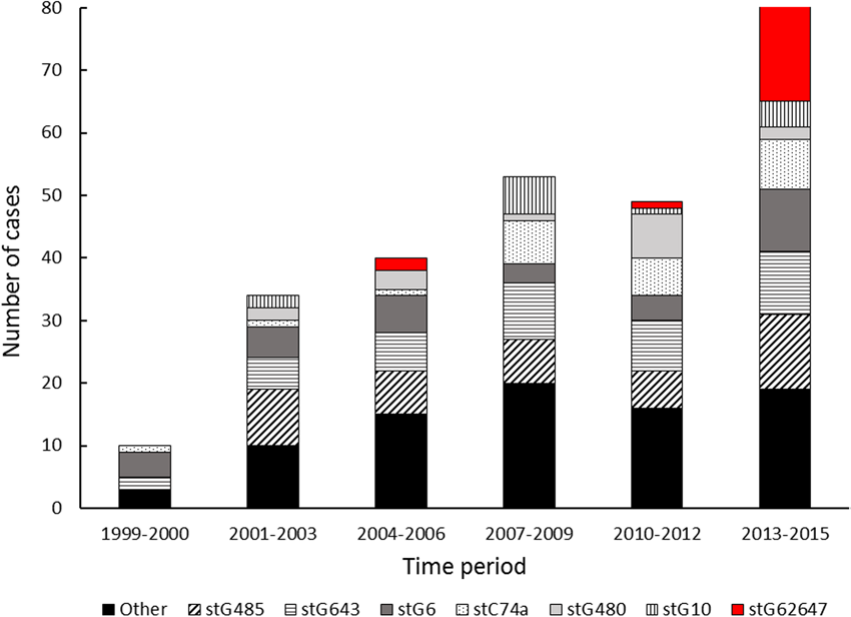
The temporal distribution of *emm*-types associated with 267 cases of invasive SDSE-disease encountered during 1999–2015. *stG62647* was the predominant *emm*-type in the period 2013–2015, comprising approximately 20% of the invasive SDSE-isolates. The category “Other” includes *stG166b* (12), *stG2078* (9), *stG652* (8), *stC5345* (4), *stC36* (2), *stC2574* (2), *stC3852* (2), *stG4831* (2), *stG5420* (2), *stC1400* (1), *stC7901* (1), *stG12* (1), *stG120* (1), *stG245* (1), *stG4222* (1), *stG507* (1), *stG653* (1), *stG6792* (1), *stG840* (1) and 29 isolates not available for *emm*-typing.

Among 267 cases of invasive SDSE disease identified from 1999 to 2015, 19 cases were attributable to group C streptococci belonging to *emm*-type *stG62647*. The first patient was a German tourist presenting with bacteraemia, acute spondylodiscitis and streptococcal toxic shock syndrome in 2006. Two additional sporadic cases were identified in 2006 and 2010; the latter was referred from another health region. However, the last 16 cases were encountered within a relatively short time frame from March 2013 to August 2015. Consequently, *stG62647* was the predominant *emm*-type during this period, accounting for >20% of all invasive SDSE-isolates. The temporal distribution of *emm*-types associated with invasive SDSE disease during 1999–2015 is depicted in Fig. 1.

The clinical characteristics of the 19 invasive infections associated with *stG62647* are presented in Table 2. Median age was 70 years, and a male predominance was noted. Osteoarticular infection was the most frequent clinical entity (42%), followed by skin and soft-tissue infection (32%). Of note, approximately half of the patients experienced severe disease manifestations: two had necrotizing soft-tissue infections, one had endocarditis, five developed streptococcal toxic shock syndrome, and three patients died within a few days after admission. Severe disease was significantly more frequent in cases associated with *stG62647* than among patients infected with SDSE-isolates belonging to other *emm-*types during the study period (47/248, 19%, *P* = 0.01).

**Table 2:** Clinical characteristics of the invasive *stG6247*-cases.

Case id.	Year	Sex	Age	SSTI	NSTI	OAI	Other	STSS	Mors30
**T267**	2006	M	70	—	—	**+**	—	**+**	—
**T274**	2006	F	93	—	—	—	**+**	—	—
**T437**	2010	F	67	—	—	**+**	—	—	—
**T560**	2013	F	87	—	—	**+**	—	—	—
**T562**	2013	M	66	—	—	**+**	—	—	—
**T576**	2013	M	28	—	**+**	—	—	**+**	—
**T599**	2013	M	60	—	—	**+**	—	—	—
**T604**	2014	M	61	—	**+**	—	—	**+**	—
**T606**	2014	M	29	—	—	**+**	—	—	—
**T607**	2014	M	86	**+**	—	—	—	—	**+**
**T619**	2014	F	63	—	—	**+**	—	**+**	—
**T630**	2014	M	77	**+**	—	—	—	**+**	—
**T642**	2015	M	70	—	—	—	**+**	—	—
**T645**	2015	M	88	**+**	—	—	—	—	**+**
**T653**	2015	F	85	**+**	—	—	—	—	—
**T654**	2015	M	63	—	—	**+**	—	—	—
**T655**	2015	F	86	—	—	—	**+**	—	**+**
**T661**	2015	M	77	**+**	—	—	—	—	—
**T666**	2015	F	72	**+**	—	—	—	—	—

Case id., case identity; SSTI, skin and soft-tissue infection; NSTI, necrotizing soft-tissue infection; OAI, osteoarticular infection; STSS, streptococcal toxic shock syndrome; Mors30, mortality within 30 days of admission; M, male; F, female. Other comprises nosocomial pneumonia (T274), prosthetic valve endocarditis (T642) and primary bacteremia (T655). Symbols + and–denote presence or absence of clinical features, respectively.

### Whole genome sequencing, MLST and SNP-analysis

**Figure 2:**
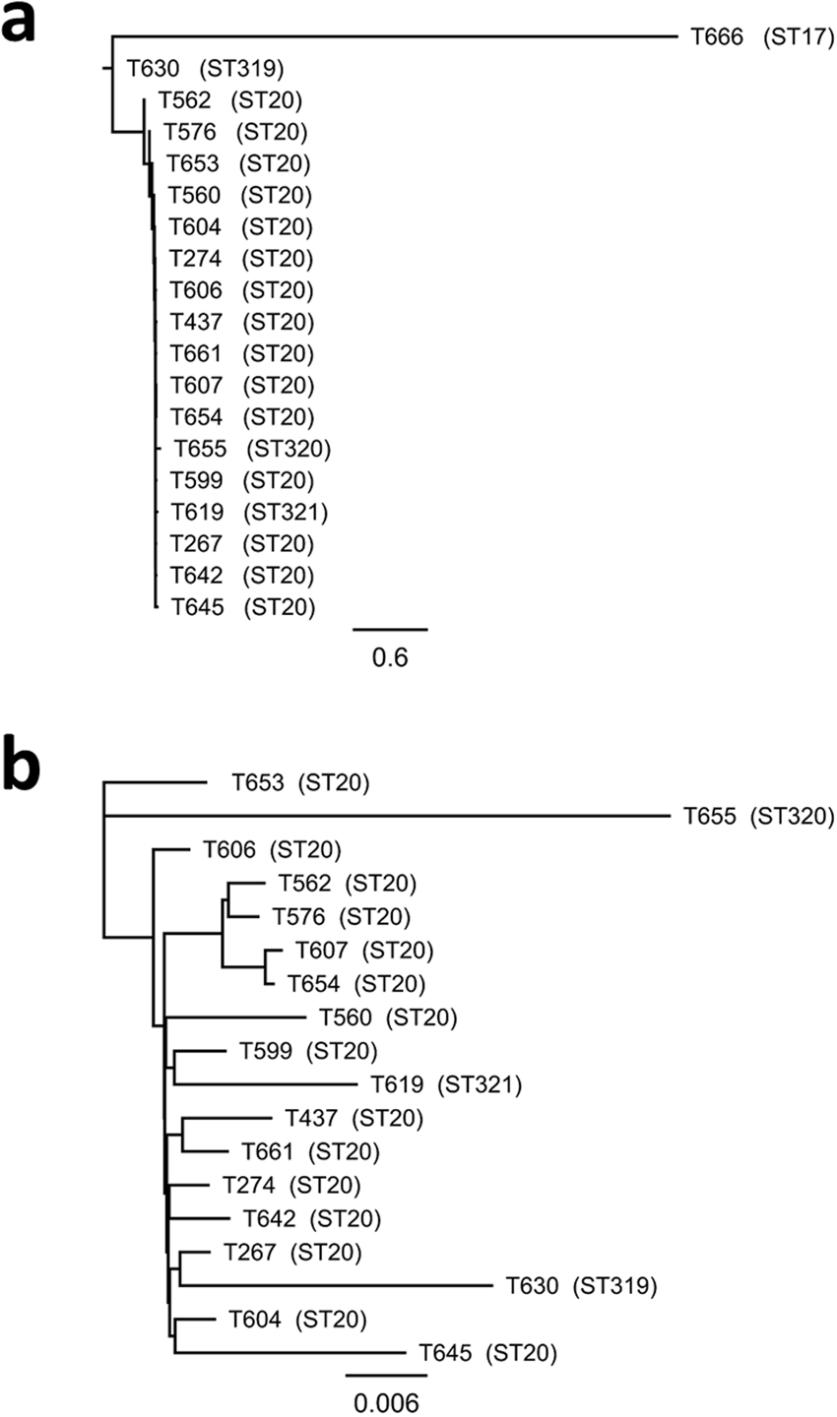
Phylogenetic tree of the *stG62647*-isolates based on core-genome SNP-analysis. (**a**) Phylogenetic tree of all 19 *stG62647*-isolates. (**b**) Phylogenetic tree of the 18 *stg62647*-isolates belonging to clonal complex 20. The ST17-isolate (T666) was omitted from the analysis to increase the phylogenetic resolution of the tree. Scales indicate substitutions per site. MLST-profile is indicated in parentheses.

The assembled genomes had an average genome-size of 2.1 Mbp, the mean coverage was ~50 fold, and the G + C-content was approximately 39%. MLST revealed a relatively uniform bacterial population, with 15 of the 19 invasive *stG62647* isolates classified as ST20. Additionally, three isolates shared six out of seven alleles with ST20, and constituted the novel ST-profiles ST319, ST320 and ST321. These 18 isolates were designated Clonal Complex 20 (CC20). The T666-isolate, however, belonged to the unrelated MLST-profile ST17, an apparently promiscuous ST-profile shared by several other SDSE *emm*-types (pubMLST.org). The *stG62647*-genomes mapped to ~90% of the SDSE ATCC 12394 reference genome, and from a core genome of 1.86 Mbp, a total of 14 163 SNPs valid in all genomes were identified. The CC20-isolates differed by an average of 214 SNPs, ranging from 16 to 718. In comparison, the T666 isolate diverged from the CC20 isolates by ~8270 SNPs. The phylogenetic tree of the 19 outbreak isolates based on core genome SNP-analysis is depicted in Fig. 2.

### Bacteriophages and CRISPRs

Bacteriophages were detected in five isolates and none of them harboured more than one phage. Four out of five had similarities to bacteriophages previously documented in *Streptococcus pyogenes*: Two isolates possessed a 35.2 kb phage displaying 67% homology to Φ315.3, one isolate contained a 41.8 kb phage bearing 70% resemblance to Φ315.2, and one isolate accommodated a 49.6 kb phage showing 60% identity to Φ315.5, all Φ315-phage-variants originally described in the *Streptococcus pyogenes emm3* isolate, MGAS315^25^. Of note, these phages have been associated with the virulence genes *mitogenic factor 4* (*mf4*), *streptococcal superantigen* (*ssa*) and *streptococcus pyogenes exotoxin A* (*speA*), respectively, but no phage-mediated virulence genes were detected in any of our isolates. The final bacteriophage consisted of a 49.6 kb element without significant similarity to previously published phages of *S. pyogenes*. CRISPR-systems were detected in all 19 *stG62647*-genomes, the majority harbouring two discrete loci. The number of CRISPR-spacers did not differ significantly between isolates containing bacteriophages (median of 14 spacers, range 11 to 23) and isolates without phages (median of 16 spacers, range 11–22), *P* = 1.0. The distribution of bacteriophages and CRISPRs is presented in Table 3.

**Table 3:** Distribution of bacteriophages and CRISPRs.

Isolate identity	ST-type	Bacteriophages	Number of CRISPR-regions	Total number of CRISPR- repeats
**T267**	ST20	—	2	15
**T274**	ST20	—	2	20
**T437**	ST20	—	2	20
**T560**	ST20	—	2	17
**T562**	ST20	—	3	19
**T576**	ST20	—	2	14
**T599**	ST20	—	3	21
**T604**	ST20	Φ315.3-like	2	11
**T606**	ST20	—	2	13
**T607**	ST20	Φ315.3-like	2	14
**T619**	ST321	—	2	22
**T630**	ST319	—	2	15
**T642**	ST20	—	3	20
**T645**	ST20	—	2	11
**T653**	ST20	—	2	11
**T654**	ST20	—	2	14
**T655**	ST320	Φ315.5-like	3	23
**T661**	ST20	Φ315.2-like	2	12
**T666**	ST17	ΦT666 - New	2	22

CRISPR, Clustered Regularly Interspaced Short Palindromic Repeats. The symbol – denotes the absence of bacteriophages.

### Virulence gene profiling

Whole genome screening of the 19 *stG62647*-isolates confirmed the presence of several established and putative virulence factors, shown in Table 1. The antiphagocytic hyaluronic acid capsule-operon has not previously been reported in SDSE, and accordingly only *hasC* was detected among our isolates. All 18 *stG62647*-isolates belonging to CC20 possessed *speG*, but lacked other differentially distributed virulence genes, such as *gfba* and *drsG*. Conversely, the ST17-isolate harboured both *gfba* and *drsG*, but lacked *speG*. No other streptococcal superantigens were identified.

A broad range of genes involved in adhesion were identified, including *dltABCD*-operon producing d-alanyl lipoteichoic acids mediating the initial contact between pathogen and host. Furthermore, two pilus-islands were discovered. These islands are also known as Fibronectin/Collagen/T-antigen-region (FCT) in *S. pyogenes*, and have been categorized into nine different FCT-regions according to their content and genomic arrangement^26^. The first pilus-island in the *stG62647*-isolates displayed a genetic structure similar to FCT-6-region, and the overall genetic identity to the FCT-6-region in the M2 *S. pyogenes*-isolate MGAS10270 (GenBank accession number CP000260) was 95%. The second pilus island did not concord with any of the nine reported FCT-regions of *S. pyogenes*. It had a structure resembling FCT-2, but had lost the terminal two transposases and the *sortaseB*-gene. However, it was structurally identical, and 95% homologous to an FCT-region found in the M58 *S. pyogenes*-strain GA40884 in GenBank (Accession number AWUA01000008).

### Virulence regulatory systems

*covRS* was conserved among all the *stG62647*-isolates, and all alleles were 100% identical. Compared to the *covRS* regulon in GGS124 (GenBank accession number AP010935), a single non-synonymous substitution in *covS* was noted, resulting in I421V.

**Figure 3:**
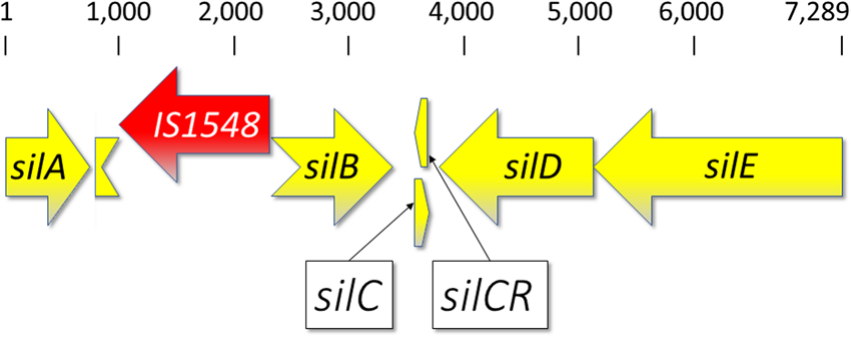
Genetic arrangement of the *streptococcal invasive locus* in the CC20-isolates. The *sil*-locus in the CC20 isolates has a transposase, IS*1548*, inserted into the *silB*-gene. *silB* encodes a sensory kinase activating the putative DNA response regulator encoded by *silA*. This leads to increased transcription of the virulence attenuating pheromone encoded by *silCR*, and suppression of the virulence associated *silC*-gene. The SilC-peptide is exported from the cell by the transporter-proteins SilD and SilE. Scale indicates nucleotide position. This disrupted sil-locus is deposited in GenBank under the accession code KY807567.

The s*treptococcal invasive locus* (*sil*) was absent in the ST17-isolate, but was detected in the remaining *stG62647*-isolates. However, in all the CC20-isolates, the *silB*-gene was truncated due to the insertion of a transposable element after nucleotide 243 (Fig. 3). The transposase was 99% homologous to IS1548, a transposable element of the IS*aS1*-family, previously reported in several beta-haemolytic streptococci^27^. To investigate if this was a common feature in SDSE, the *sil*-locus in the 23 SDSE-genomes currently in Genbank were examined (ncbi.nlm.nih.gov/genome). None of them contained a transposase within the *silB*-gene, nor was this identified in any of the 333*S. pyogenes*-genomes deposited in GenBank. Lastly, from our sample of 267 invasive SDSE isolates identified between 1999 and 2015, an additional 76 isolates, chosen to represent the diversity of clinical and molecular characteristics, have been subjected to whole genome sequencing (Unpublished data). A transposable element within the *silB*-gene was not detected in any of these genomes, indicating that this is not a common genetic feature of SDSE-isolates within our geographical confines.

## Discussion

*Streptococcus dysgalactiae* subspecies *equisimilis* (SDSE) is increasingly implicated in invasive infections worldwide, but the evolutionary events underlying this upsurge are unknown. With the present work, we have documented the recent emergence of a virulent SDSE genotype, *stG62647*, in western Norway, and delineated its clinical and genetic characteristics. To the best of our knowledge, this is the first study employing large scale whole genome sequencing to explore the genetic landscape of epidemic lineages in SDSE. Wang *et al*. characterized an outbreak of tonsillopharyngitis caused by SDSE in Hong Kong, but they sequenced only one isolate^28^.

*stG62647*-isolates did not cause invasive SDSE-infections in our region until a few sporadic cases were encountered from 2006. However, in the period from March 2013 to August 2015 it became the predominant *emm*-type in our health-region, responsible for more than 20% of all invasive SDSE-disease, and comprising 80% of the invasive group C streptococcal-isolates. Although this *emm*-type has previously been reported from several countries and continents, its relative proportion has generally been below 5%, indicating mainly sporadic encounters^8, 29, 30^. Interestingly, in a recent study from our neighbouring country, Sweden, *stG62647* was one of the most frequently occurring *emm*-types during 2008–2011, comprising 25% of the invasive SDSE-isolates identified^31^. Differently, Rantala *et al*. found only one *stG62647*-isolate among 140 invasive SDSE cases encountered in Finland between 1995 and 2004^32^. Taken together, this might indicate that the emergence of a virulent *stG62647*-type in the Nordic countries is a fairly recent event.

Based on MLST data, the *stG62647*-isolates constituted a relatively homogenous group, with 15 out of 19 belonging to ST20, and 18 out of 19 grouped together as CC20. However, SNP-analysis revealed a more diverse population, emphasizing the superior epidemiological resolution of a SNP-analysis compared to traditional methods. Moreover, it suggests that the genetic events resulting in the epidemiological spread of this genotype have occurred some time ago. The expected rates of spontaneous mutations and recombination events in SDSE have not been well defined, though, making conclusions on evolutionary relationships and clonality difficult.

Notably, half the patients in our study infected with *stG62647*-isolates experienced a fulminant clinical course, including streptococcal toxic shock syndrome and necrotizing soft-tissue infections. Such disease manifestations are usually associated with *S. pyogenes*, although our findings are in accordance with previous reports on *stG62647*. In Sweden, this *emm*-type was significantly associated with an invasive phenotype, and in a recent report from Austria *stG62647* was linked to severe clinical manifestations^31, 33^. However, most epidemiological reports on SDSE genotypes comprise too few *stG62647*-isolates to evaluate its correlation with disease severity.

The *streptococcal invasive locus* (*sil*) has been demonstrated to repress bacterial virulence through the production of the pheromone like peptide silCR^13^. This locus was initially described in an M14 *S. pyogenes* isolate procured from a patient with necrotizing soft-tissue infections, and the strain was discovered to have a missense-mutation in the *silCR* start-codon^13^. Subsequent experimental murine skin-infection models revealed these *silCR*-mutants to confer increased mortality and extensive skin necrosis, whereas concomitant administration of synthetic silCR resulted in attenuated virulence^13, 34^. Surprisingly, all our CC20-isolates harboured a truncated *silB*-allele due to the insertion of an IS1548-transposon. This feature appears to be rare, and was neither identified among any of the SDSE- or *S. pyogenes*-genomes currently available in GenBank, nor in the genomes of contemporary invasive SDSE isolates from our health region. The *silB*-gene encodes a sensory kinase that activates the transcriptional regulator *silA*, leading to an upregulation of silCR-production and exportation, and concurrent suppression of *silC* ^15, 35^. In a recent murine model study, SDSE grown in the presence of silCR caused a milder disease than the control group. Inversely, mice vaccinated against silCR developed a significantly more severe illness than non-vaccinated mice^6^. Although not experimentally verified, it is plausible that the lack of silB in our *stG62647*-isolates would impair the silCR-levels, and favour the production of the virulence associated silC-peptide. This would likely constitute a significant contribution to the aggressive phenotype associated with our *stG62647*-isolates belonging to CC20. Experimentally induced mutations in *silB* in M14 *S. pyogenes*-strains resulted in reduced virulence^13, 36^. However, those isolates concurrently contained a truncated *silCR*-gene, and the results are thus not readily transferable.

IS1548 is a transposable element of the ISA*S1*-family, initially described in invasive *Streptococcus agalactiae* strains, but subsequently also detected in *S. pyogenes* and SDSE^27, 37^. It encodes two putative proteins of 377 and 62 amino acids, respectively, and has 19 bp imperfect inverted repeats at its termini. Interestingly, IS1548 has been reported to modulate the expression of several virulence factors in *Streptococcus agalactiae*. Al Safadi *et al*. demonstrated that the insertion of IS1548 immediately upstream of the laminin binding gene *lmb*, was associated with an ~5-fold increase both in the transcription of *lmb*, and in the phenotypic bacterial adhesion to laminin^38^. Conversely, insertion of IS1548 into the *hylB* and *cpsD* genes, abrogated the production of hyaluronidase and bacterial capsule, respectively^37, 39^. Of note, IS1548 has also been documented in the *covRS*-system in *S. pyogenes*. Several *S. pyogenes*-strains were passed through an experimental murine model, and spontaneous insertion of IS1548 into the *covS*-gene was observed in two isolates. These genetic alterations in the *covRS*-system rendered the bacteria more virulent and led to more extensive necrotic skin lesions^12^. Although mutational transition to a more virulent phenotype undoubtedly renders the bacteria more capable of invasive disease, it does not necessarily represent an evolutionary advantage. Trevino *et al*. showed that highly virulent *covRS*-mutated *S. pyogenes*-isolates indeed were significantly outcompeted by wild-type strains during growth in human saliva, indicating that different phenotypic properties might be required for a commensal state in the upper respiratory tract^14^. IS1548 has been suggested to mediate adaptive mutations, allowing the bacteria to switch between pathogenicity and commensalism^27^. Insertion of a transposable element is conceivably a more dynamic evolutionary transition than adaptation through point-mutations and deletions. We speculate that the insertion of IS1548 into the *silB*-gene confers a more aggressive and invasive bacterial phenotype through the downregulation of *silCR*. Furthermore, it is plausible that the process is reversible under certain environmental conditions, facilitating the return to a commensal state. The pathogenic mechanisms and bacterial signalling involved in this hypothesized transition have yet to be explored, and deserves further investigation.

A genetic dissection of the *stG62647*-isolates revealed the presence of several important virulence factors, including genes involved in immune evasion (*emm*, *protein G* and *c*5*a-peptidase*), adhesion (*dltABCD*, *fbp54*, *gapC*, *shr* and *lmb*), tissue penetration (*streptokinase*) and toxins (*streptolysin O* and *streptolysin S*). However, these genes appear to be ubiquitous in SDSE, and are thus unlikely to have a pivotal role in the successful epidemic spread and observed disease severity of *stG62647* ^5^. The differentially distributed *speG* was identified in all the CC20-isolates. *speG* does not confer superantigen-activity, though, and a correlation with disease severity has not been demonstrated^40, 41^. The *covRS*-regulon was conserved among the *stG62647*-isolates, and all showed a single amino acid substitution in covS, I421V, as compared to GGS124. However, a valine residue in this position is also present in wild type *S. pyogenes*-isolates, thus unlikely to be of clinical significance^42^.

Although bacteriophages were encountered in a subset of the isolates, no established or putative phage-associated virulence genes were detected. The CRISPR-cas system constitutes a part of the adaptive immune system in prokaryotic cells, conferring resistance to mobile genetic elements and bacteriophages^43^. Previous encounters with bacteriophages are deposited in the CRIPSR-locus as short sequence-specific DNA-fragments termed spacers. For *S. pyogenes*, an inverse relationship between the number of spacers and susceptibility to bacteriophages has been reported^43^. We did not observe such an association among our SDSE-isolates, albeit the frequency of bacteriophages was very low. We found our SDSE-isolates to contain markedly fewer bacteriophages and a higher number of spacers than *S. pyogenes*-genomes currently deposited in GenBank. Similar observations were reported by Shimomura *et al*., and a CRISPR-mediated increased resistance to bacteriophage-infection among SDSE compared to *S. pyogenes* is conceivable^44^.

The present study has some limitations. First, the partial retrospective design increased the risk of diagnostic misclassification, potentially leading to the underestimation of invasive SDSE and *stG62647*-infections. However, a scrutinous review of medical records likely led to the inclusion of all relevant SDSE cases in our region during the study period. Secondly, we acknowledge that the rise and fall of epidemic lineages of microbes is multifactorial, and although bacterial genetic events undoubtedly are contributory, knowledge on the prevalence of the outbreak genotype in asymptomatic carriers, along with the protective specific immunity in our population during the study period, would have increased our understanding of this *stG62647*-outbreak. Lastly, several of our proposed pathogenic mechanisms are based on extrapolation of experimental data from closely related species, as knowledge on virulence properties in SDSE is scarce. A substantial fraction of the genetic repertoire specific for the *stG62647*-lineage encodes hypothetical proteins, and their pathogenic contribution is difficult to ascertain within the confines of current knowledge. Hence, the possibility that the observed virulence potential of the *stG62647*-isolates emanates from any of these genetic elements cannot be ruled out. Furthermore, the significance of an insertional element in the *silB*-gene has not been verified experimentally, and its contribution to disease invasiveness or microenvironmental adaptation has yet to be elucidated. Ideally, the *sil*-locus should be examined from *stG62647*-isolates collected from different ecological niches, including asymptomatic carriers. A final conclusion could be obtained after the deletion of *IS*1548 from the *silB* gene of one of the *stG62647-*strain and the comparison of the virulence of the deletion mutant and the wild-type strain in an animal model. Adaptive virulence regulation through insertional elements constitutes an intriguing concept, and further studies are warranted.

## Conclusions

We report the emergence of a novel and virulent SDSE genotype, *stG62647*, in our health region. The infections associated with *stG62647* appeared to pursue a severe clinical course, including the occurrence of streptococcal toxic shock syndrome, necrotizing soft-tissue infections and endocarditis. We speculate that its virulence could be partly attributable to the insertion of a transposable element, IS1548, into the *streptococcal invasive locus* (*sil*), thus abrogating the virulence attenuating control exerted by this regulon. Invasive SDSE disease is currently three times more common than invasive *S. pyogenes* infections in our region, and sustained epidemiological attentiveness is indicated.

### Ethical considerations

The study underwent institutional ethics review and approval (2010/1406 Regional Ethics Committee West, Norway). Informed consent was obtained from participants. All experiments were carried out in full accordance with the approved ethics applications specified above.

### Availability of data and materials

The genomes of the *stG62647*-isolates have been deposited in the SRA GenBank database under the study accession number SRP103012 (www.ncbi.nlm.nih.gov/sra). Isolate T642 has been deposited at DDBJ/ENA/GenBank under the accession NBUZ00000000, the version described in this paper is version NBUZ01000000. The *streptococcal invasive locus* containing a truncated *silB*-gene due to the insertion of IS1548, is deposited in GenBank under the accession number KY807567.
